# Sex-specific seasonal variation in home range size in a sedentary avian predator

**DOI:** 10.1007/s00442-025-05812-2

**Published:** 2025-10-14

**Authors:** Ülo Väli, Jaan Grosberg, Paweł Mirski

**Affiliations:** 1 Institute of Agricultural and Environmental Sciences, Estonian University of Life Sciences, Kreutzwaldi 5D, 51006 Tartu, Estonia; 2 Faculty of Biology, University of Białystok, Ciołkowskiego 1J, 15-245 Białystok, Poland

**Keywords:** Goshawk, GPS tracking, Intrapair competition, Movement ecology, Sexual conflict

## Abstract

Spatial ecology of species is determined by the interplay of various social and demographic factors. Variation in utilisation of space may also reduce the conflict between individuals competing for the same resources. We simultaneously evaluated the effects of sex, territoriality and reproductive performance on the space use of the Eurasian goshawk, *Accipiter gentilis*, by analysing the home range sizes of 25 individuals across various stages of the annual cycle for up to 7 years. The mean annual home range (90% autocorrelated kernel density estimate) of territorial goshawks was slightly smaller than that of non-territorial hawks, and the ranges of successful birds tended to be smaller than those of non-successful territorial goshawks. The median home range of territorial females (295 km^2^) was five times larger than that of males (58 km^2^). While the home ranges of males overlapped by 89–100% with those of females, only 7–14% of females’ ranges were utilised by their male counterparts. The home ranges of territorial females gradually increased after the incubation stage, and their fidelity to nest sites weakened. Males maintained their association with their nest sites throughout the year. These sex-specific differences may be attributed to distinct strategies, with males acting as territory holders and females exploring potential mates and nest sites, or to the reduction of competition between partners. The findings underscore the significance of long-term tracking across annual cycles in determining annual home ranges and show how complex space use patterns are in sedentary dietary specialists under seasonally varying food resources and intra- and extrapair competition.

**Supplementary Information:**

The online version contains supplementary material available at 10.1007/s00442-025-05812-2.

## Introduction

Movement is a key feature for animals, steering all their activities. At the individual level, the concept of home range occupies a central position in movement ecology (Powell 2000). Originally defined as the area used by the individual to carry out its normal life activities, such as foraging, mating and caring for its offspring (Burt 1943), this definition continues to be widely employed today (Alston et al. 2022). However, the term is now more commonly applied to any areas used annually or seasonally, rather than being specific to breeding activities (Kenward 2006; Powell 2000; Powell and Mitchell 2012). The identification of factors influencing the home range holds paramount importance for unravelling the territorial behaviour of individuals and their interactions with each other and environmental determinants (Börger 2016; Williams et al. 2020; Nathan et al. 2022).

In solitarily breeding species, the breeding home range comprises both the breeding territory and the surrounding foraging area (Newton 1998). The breeding territory is an area that individuals defend not only from potential predators but also from conspecifics (Block and Brennan 1993). However, it is primarily the foraging area that determines the size of the home range through the extent of suitable habitat or prey availability (Newton 1998; Rolando 2002). Individual home ranges are not necessarily mutually exclusive, resulting in potential competition between individuals (McLoughin et al. 2000; Bosch et al. 2010; Krupiński et al. 2021), because they are usually limited by food availability (Martin 1987). Although individuals naturally aim to diminish the competition, it is often unavoidable. For example, if both members of a breeding pair are responsible for searching for food for their offspring, they are competing with each other for space and resources (Newton 1998). In sexually dimorphic species, males and females may use different resources, reducing competition and increasing prey availability for parents (Gorell et al. 2005). Furthermore, males and females may respond differently to seasonal changes (Belthoff et al. 1993); this stresses the need to evaluate not only potential overlap of home ranges and food composition but also interactions between various variables and seasons.

Home range size is also impacted by the reproductive success and status of an individual. For example, home range may change following breeding failure or depend on the decision to skip breeding (Rolando 2002). Furthermore, populations of long-lived species often include non-territorial individuals not attached to any nest site, thus free from restrictions imposed by breeding (Penteriani et al. 2011; Rolando 2002). These individuals may be adults unable to achieve a territory; however, immatures pass through this stage nearly exclusively. The home ranges of non-territorial individuals may or may not resemble those of territorial ones, but they unquestionably warrant special research attention, because non-breeders often represent the future potential of the population (Newton 1998; Tanferna et al. 2013). Obviously, territorial (thus potentially breeding) birds and non-territorial birds differ in their behaviour, needs and limitations; thus, comparison of their home ranges is essential to obtain a complete picture of space use in animal populations.

The Eurasian goshawk, *Accipiter gentilis* (hereafter goshawk), is a widely distributed bird of prey that breeds across the Palaearctic forest zone (Kenward 2006; BirdLife International 2021). The goshawk exhibits distinct sexual dimorphism, with females significantly larger than males. The roles of females and males during the early breeding stages differ markedly, with females responsible for incubating and caring for nestlings, while males engage in hunting and providing food to the nest. Later in the breeding cycle, both sexes partake in hunting activities (Kenward 2006). This sexual segregation contributes to observed differences in home ranges. Telemetry studies on Eurasian goshawks, as well as closely related American goshawks, *Accipiter atricapillus*, have consistently shown that the home ranges of females tend to be smaller than those of males (Hargys et al. 1994; Iverson et al. 1996; Tornberg and Colpaert 2001; Tornberg et al. 2006; Moser and Garton 2019; Blakey et al. 2020). However, remarkable variation may occur over seasons (Iverson et al. 1996; Boal et al. 2003; Moser and Garton 2019; Blakey et al. 2020), and contrasting patterns have also been identified (e.g. Boal et al. 2003; Tornberg et al. 2006). While numerous studies have explored home ranges in North America in recent decades (e.g. Hargys et al. 1994; Iverson et al. 1996; Boal et al. 2003; Moser and Garton 2019; Blakey et al. 2020), only a limited number have been conducted in Europe after pioneering radiotracking studies in the 1980s (Kenward et al. 1981; Kenward 1982; Widen 1985; Tornberg and Colpaert 2001; Rutz 2006). The latter is surprising, given the widespread distribution of the species across the continent, and notably, none of these studies on Eurasian goshawks have utilised precise GPS-based tracking.

Hence, the goshawk, a (mostly) territorial, sedentary, long-lived raptor with pronounced sexual dimorphism, is a good model for studying important questions in home range ecology, such as the reduction of conflict between sexes competing for the same resources over the resource-varying seasons and the role of territoriality in spatial requirements. In this study, we addressed questions on annual dynamics, the impact of sex and breeding status on space use demands by conducting GPS tracking on goshawk individuals tracked for up to 7 years in Estonia, northern Europe. Our hypotheses were as follows: H1: home ranges of territorial birds, associated with specific breeding territories, are smaller than those of non-territorial birds that are diminishing competition by travelling longer distances, which leads to larger home ranges. Alternatively, non-territorial birds may settle in food-rich sites, not defended by territorial birds, and thus exhibit smaller home ranges. H2: Home ranges of successfully breeding birds are smaller than those of non-breeding or unsuccessfully breeding birds. This may result from the tight association with the nest, but also from the high quality of habitats, where abundant resources enable successful breeding. However, alternatively, the latter group of birds, lacking the offspring and thus having lower pressure to hunt, may use a smaller range than those collecting food for their young. H3: Both breeding males and females gradually increase their home ranges during the annual cycle (incubation time < nestlings in nest < fledglings near nest < non-breeding) along with the increasing food demands of young and decreasing association of adults with nests. The temporal change is more pronounced in females who, unlike males, are tightly associated with nests during incubation time, and require larger (and scarcer) prey to feed themselves during the non-breeding stage. In addition, we tested whether reproductive performance influences temporal changes in home range size. H4: To decrease competition between partners, home ranges of males and females overlap less in the non-breeding season compared with other stages of the annual cycle. Given that the overlap was possible to calculate directly only for pairs tracked simultaneously, H4 was also evaluated indirectly by measuring distances between locations of all studied birds and their nests throughout the annual cycle.

## Materials and methods

### The study population

**Fig. 1:**
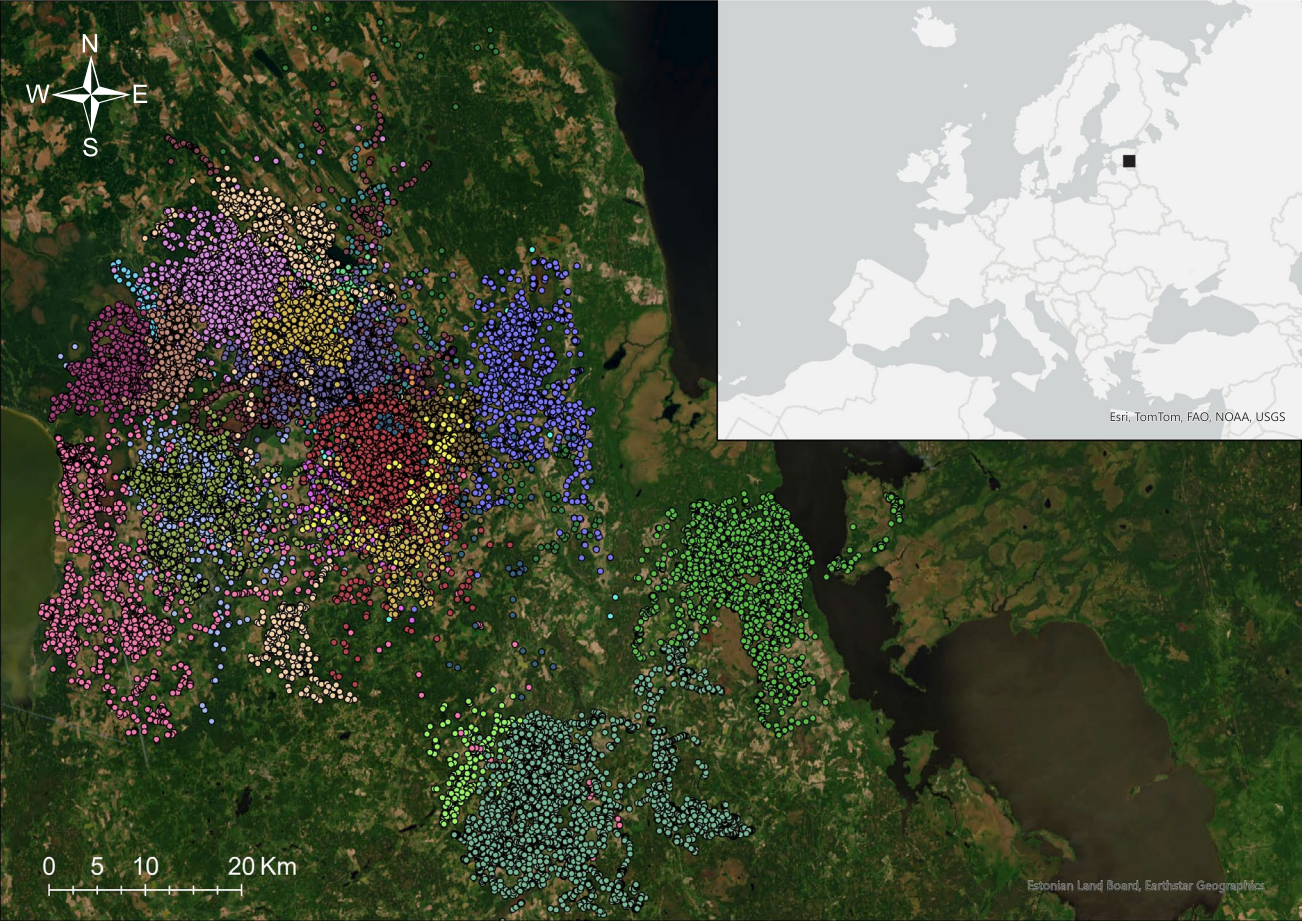
Spatial distribution of the GPS fixes of the studied goshawks; different colours represent different individuals (*n* = 25). In the top right corner, the location of the study area in Europe is shown

The study was conducted in Estonia, northern Europe (Fig. 1), where the nominate subspecies *Accipiter gentilis gentilis* is found (Kenward 2006). Estonia is a flat lowland country situated in the hemiboreal vegetation zone (Peltonen-Sainio 2012). The climate is characterised by temperate and mild conditions, featuring warm summers and relatively severe winters (Estonian Environment Agency 2025). Typically, the coldest month is February, with an average daily air temperature of − 5 °C, while the warmest month is July, with an average temperature of + 18 °C (Estonian Environment Agency 2025).

The goshawks were tracked in the south-eastern part of Estonia, near Tartu, in an area of approximately 5300 km^2^ (58°20´ N, 26°40´ E, Fig. 1). The study area is a landscape mosaic composed of forests, farmlands, and wetlands of various sizes, with few towns and relatively small roads. The predominant land cover types include woodlands (49.5%) and arable fields (29.1%), with permanent grasslands (9.3%), wetlands (5.1%), water bodies (2.2%), and other habitats present in smaller proportions (Land Board Geoportal 2022).

There are approximately 400–600 pairs of goshawks breeding in Estonia; the abundance was at least twofold higher in the late twentieth century (Elts et al. 2019, Väli et al. 2019). Consequently, the goshawk is considered a threatened species and is included in the list of protected animals in Estonia. Adult goshawks in Estonia are sedentary, while juveniles exhibit partial migration (Väli and Vainu 2013). According to Krüger (2005), 42% of females start to breed in the second calendar year (2cy). Although some 2cy males can breed too, the vast majority start breeding later. This pattern is valid also in Estonia (personal observations of authors). Breeding activities commence with the construction and renovation of nests in March (sometimes in February or April); eggs are typically laid in early or mid-April, but female stays close to the nest already earlier; the incubation stage lasts approximately 40 days, leading to the hatching of offspring in mid-May; nestlings remain in the nest for 35–42 days and fledge in late June or early July; the young birds stay in proximity to the nest until mid-August, after which they disperse (Väli 2018).

Although the goshawk is primarily a forest-dwelling species (Kenward 1996; Kenward and Widén 1989), it demonstrates adaptability by thriving also in mosaic agricultural landscapes (Kenward 1982, Selås et al. 2008, Kudo et al. 2005) and has even recently colonised urban areas (Kenward 2006; Rutz 2006, 2008; Väli et al. 2023). As a central-place forager, it typically breeds solitarily and is evenly distributed across landscapes (Kenward 1982; Widén 1985; Tornberg and Colpaert 2001; Kudo et al. 2005; Väli et al. 2024b).

The goshawk preys upon various avian and mammal species, with a primary focus on medium-sized birds (Kenward 2006; Rutz et al. 2006; Tornberg et al. 2006). In Estonia, 98% of the diet consists of birds (Väli et al. 2024a), particularly medium-sized species such as feral pigeons (*Columba livia domestica*), wood pigeons (*Columba palumbus*), corvids (*Corvidae*), and thrushes (*Turdus*; Lõhmus 1993, Väli et al. 2024a). Given the clear size difference between sexes (Estonian males weigh 700–1020 g, and females 1100–1760 g; Kumari 1954), the sexes probably focus on different prey items, although the confirming regional studies are currently lacking. In addition, the winter diet remains unstudied in Estonia, but in nearby Finland and Sweden, an increase in the share of mammals in the diet during winter has been observed (Kenward et al. 1981; Widén 1987, Tornberg, Colpaert 2001).

### Tracking of birds

A total of 25 goshawks were studied (Supplementary Table 1, Väli et al. 2025). In total, 53 goshawk-years were included in the analysis. The tracking duration varied due to the diverse lifespan of birds and transmitters, ranging from 31 to 2355 days (i.e. up to 7 years; see Supplementary Table 1), with an average tracking period of 543 ± 559 (SD) days.

The sample comprised 14 males and 11 females, including four breeding pairs. Among the tracked birds, 21 were territorial, while 4 individuals showed no association with a specific nest and did not exhibit nesting behaviour. Within the group of territorial birds, we differentiated between successful breeders (those with at least one fledged offspring in a given ‘goshawk-year’) and unsuccessful individuals (out of seven studied cases, four nests were ‘decorated’ but eggs were not laid; once, the female stopped incubation after 10 days, and twice, small nestlings perished). Notably, the reproductive performance of individual birds was usually consistent over the years, i.e. successful birds were successful in all studied years, and vice versa (Supplementary Table 1). Initially, we categorised second-calendar-year (2cy) birds separately, but since all three 2cy females were associated with a nest and breeding, they were included in the group of territorial birds. The two 2cy males, however, were non-territorial. All individuals but one adult female stayed in the study area. We excluded the movements of this bird (191324_5) during her repeated long-distance excursions to North-Western Russia (60°20´ N, 29°40´ E) on 30-Oct-2022–28-Feb-2023 and 23-Nov-2023–09-Feb-2024 (her last known location), considering these as outliers due to being the only instances of such extensive exploratory (migratory) movements. Given the potential of migratory origin and long-distance exploratory movements, no 1cy birds were included in the analysis.

The goshawks were equipped with solar-powered GPS transmitters weighing between 15 and 30 g (< 3% of body weight) provided by Ornitela UAB, Vilnius. These transmitters, attached as backpacks using full Teflon harnesses, transmitted data via the mobile communication network. Previous research has shown that backpack-mounted transmitters have no survival impact on adult goshawks (Reynolds et al. 2004). The transmitters were equipped with remote programming capabilities, allowing us to adjust settings based on the battery’s charging status. Typically, GPS fixes were collected every 10 min from sunrise to sunset. However, when the transmitter’s battery charge fell below 25%, especially in winter and during female incubation, the frequency was reduced to 1 to 4 h. In total, 361,230 locations were included in the analysis.

The sizes of the home ranges were assessed using data collected throughout the entire year, as well as during specific stages of the annual cycle. For the latter, the year was divided into four distinct stages: (1) laying and incubation stage (01-Apr–31-May; 61 days); (2) stage of nestlings at nest (01-Jun–15-Jul; 45 days); (3) time of the fledglings spending near nests (16-Jul–31-Aug; 47 days); (4) non-breeding stage (01-Sep–31-Mar; 212 days). The determination of breeding stages drew upon both the breeding phenology outlined in the literature sources (Kenward 2006; Väli 2018) and the observed behaviour of the specimens in this study (Supplementary Fig. 1). The tracked females (*n* = 8), on average, started (preparation for) laying (i.e. stayed short periods in horizontal posture) on 4-Apr (± 5.9 days SD) and started continuous incubation on 9-Apr (± 6.7 days); they accomplished incubation (stood on nest) on 26-May (± 5.4 days), on average. Hence, our generalised approach enabling comparisons between groups would not have been different from a methodologically more limited individual approach by categorising stages uniquely for each bird by accelerometer data. Notably, the accelerometer indicates (with some uncertainty) by posture clearly only the incubation stage of breeding females but no other stages (Supplementary Fig. 1). Hence, such an individual approach cannot be applied in comparison of various stages of breeders and non-breeders, as well as successful and unsuccessful breeders. Yet, accelerometer data and information on nest attendance were used to confirm breeding/non-breeding status of individuals and to support the dates upon which the annual cycle was divided into stages.

### Estimating home range size

Our dataset included data collected at different sampling regimes, depending on seasonally changing battery charging potential. Data collected with relatively short intervals during spring and summer might be affected by autocorrelation issues; therefore, we chose the home range estimation method, which is based on effective and acknowledged utilisation distribution methodology, but also accounts for temporal autocorrelation. Home range sizes were calculated using the autocorrelated kernel density estimation method (AKDE; Fleming et al. 2015), which fits several candidate continuous-time movement models to each movement trajectory to estimate the underlying autocorrelation structure and then selects the most parsimonious model using AICc before estimating the utilisation distribution. Home range size was calculated from the 90% AKDE isopleth using the local geographic projection (EST97). Calculations were done in the *amt* package (Signer et al. 2019) in R software (version 4.3.2; R Core Team 2023). We also calculated the overlap between home ranges (the proportion which the home range of one individual covers in the home range of another individual) of males and females in breeding pairs that were tracked simultaneously. ArcGIS Pro 3.0.0 (ESRI 2022) tool *Count Overlapping Features* was employed to assess this overlap, and the tool Generate near table was used to measure distances between GPS fixes and nests of the tracked goshawks.

### Statistical analysis

Statistical analysis was conducted using R (version 4.3.2; R Core Team 2023). Mean home range sizes of studied groups were characterised using mean annual values for each individual. However, interannual variation of individual home ranges was considered in between-group comparisons, which were tested using linear mixed models (LMM, package *lme4 v 1.1-35.1*; Bates et al. 2015). In LMMs, home range size served as a continuous independent factor (log-transformed to normalise the distribution of errors), with sex, territoriality, reproductive performance (successfully breeding or unsuccessful individual), or stage of the annual cycle as categorical predictors. Individual identity was always included as a random factor. Given the small and unbalanced sample, and the focus on hypothesis testing rather than variable selection, each categorical predictor was analysed in a separate model. Likelihood-ratio tests with Chi-square approximation were employed to assess the significance of the models compared to null models (models with only a random factor). To evaluate home range size differences between particular stages within each sex, or among successful and unsuccessful birds, pairwise post hoc tests with Tukey adjustments for multiple comparisons were used in the package *emmeans v 1.10.4* (Lenth 2024). The package *sjPlot v 2.8.15* (Lüdecke 2023) was utilised to estimate marginal *R*^*2*^ and conditional *R*^*2*^, describing variances explained by only fixed factors and by both fixed and random factors, respectively (Nakagawa et al. 2017).

For testing differences between sexes throughout the annual cycle, a generalised additive mixed model (GAMM) was applied using the package *mgcv v. 1.9–1* (Wood 2011). The log-transformed shortest distance between a GPS fix and the individual’s nest used in a given year (starting from incubation time) was a dependent variable; sex was included as a categorical predictor and day of year as a smooth term (*k* = 7, bs = ‘cs’); individual identity and year were included as random effects (bs = ‘re’).

## Results

**Fig. 2:**
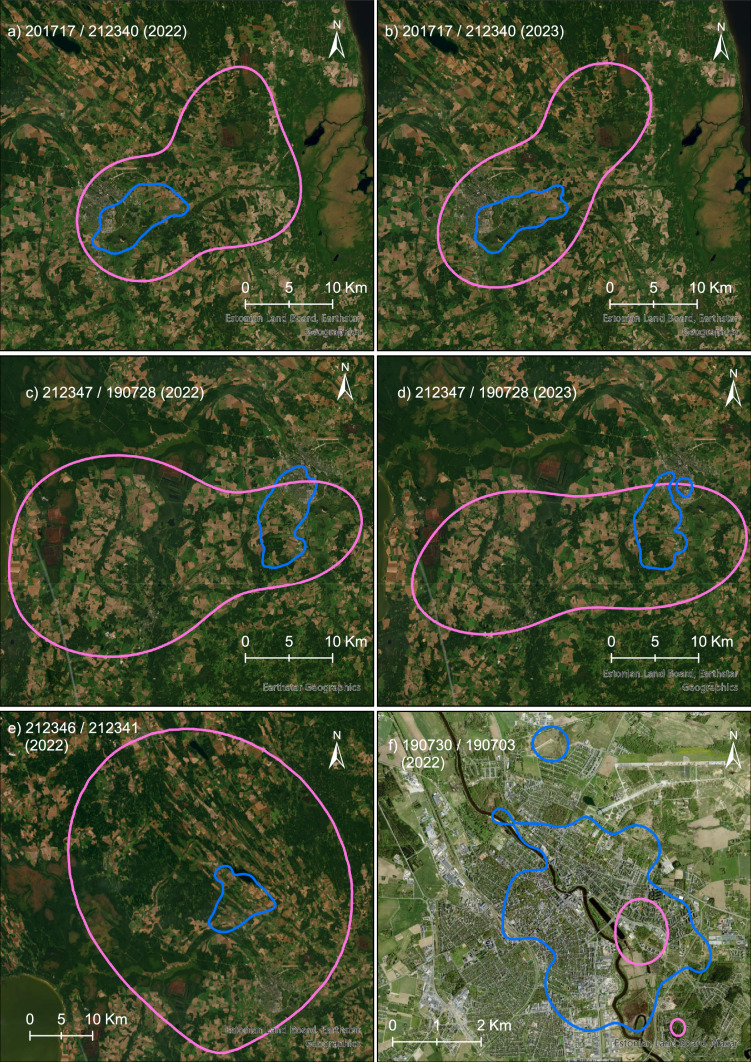
Annual home ranges (90% AKDE) of the tracked goshawk pairs. Note that birds with home ranges presented in **a** and** b**, similar to those in **c** and **d**, represent pairs tracked for 2 years, respectively. Male at **e** and female at **f** were tracked only during incubation (23.03–22.04) or incubation and nestling stage (23.03–30.06), respectively

Prior to testing particular hypotheses, yearly home ranges of males and females were compared, and the consistency of home range sizes was evaluated. The yearly mean home range (90% AKDE) of territorial females (499.1 ± 594.1 (SD) km^2^, *n* = 10) was more than nine times larger than that of males (54.4 ± 31.4 km^2^, *n* = 11; LMM: *χ*^*2*^ = 4.12, *P* = 0.042); the size difference of median home ranges (294.9 km^2^ in females, 58.3 km^2^ in males) was fivefold. However, there was extensive variation between individuals (especially females), as indicated by the remarkably higher conditional *R*^*2*^ (0.96) compared to the marginal *R*^*2*^ (0.14). In the two pairs studied for two full years, home ranges of males overlapped by 89–100% with those of females, while those of females overlapped 7–14% with males’ home ranges (Fig. 2, Supplementary Table 2, see also Supplementary Video 1). The home ranges showed persistence over the years, best illustrated by the two males tracked for 5 and 7 years, respectively (Supplementary Fig. 2, see also Fig. 2).

The testing of H1 showed that the home ranges of non-territorial individuals were significantly larger (665.9 ± 815.5 km^2^, *n* = 4 individuals) than those of territorial ones (266.1 ± 459.4 km^2^, n = 21), but the difference was not significant (*χ*^*2*^ = 2.69, *P* = 0.101). Testing of the H2 revealed that, on average, the annual home ranges of successful territorial birds tended to be smaller than those of unsuccessful territorial birds, but these differences were not significant (377.9 ± 482.8 km^2^ (*n* = 7) and 781.6 ± 847.1 km^2^ (*n* = 3) for females, *χ*^*2*^ = 0.75, *P* = 0.384; and 45.6 ± 27.8 km^2^ (*n* = 6) and 64.9 ± 35.8 km^2^ (*n* = 5) for males, *χ*^*2*^ = 0.82, *P* = 0.364, respectively).

**Fig. 3:**
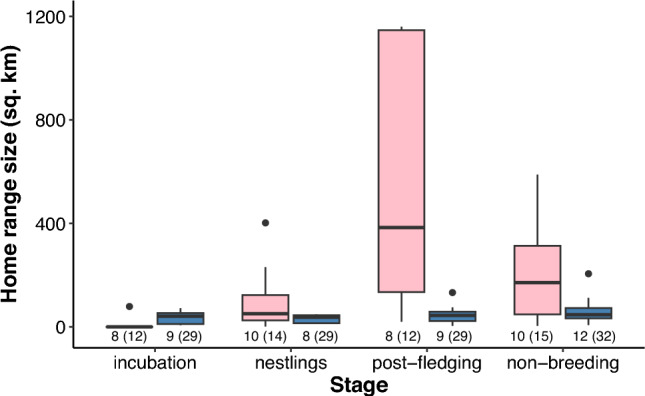
Home range sizes (90% AKDE) of female (pink) and male (blue) goshawks during different breeding stages and the non-breeding stage. The bold line indicates the median, the box shows quartiles, the whiskers indicate the extreme data points within 1.5 × the interquartile range from the quartile boundaries and dots are data points beyond that range. Numbers of studied individuals (and goshawk-years in brackets) are presented on the x-axis

To test the H3, home ranges at the four stages of the annual cycle were compared. In females, the home range size changed significantly during the four breeding stages (*χ*^*2*^ = 58.1, *P* < 0.001) as the home ranges during the incubation time were significantly smaller than those during the nestling, post-fledging, and non-breeding stages (post hoc tests: *P* < 0.001; Fig. 3, Supplementary Video 1). Home ranges at the post-fledging stage tended to be larger than those during the nestling and non-breeding stages (*P* = 0.093 both), with no difference between the latter two (*P* = 1.0). Home ranges of unsuccessful females were slightly larger during incubation time than those of the successful females, but similar during the nestling stage; however, home ranges of unsuccessful females tended to be larger than those of successful females in post-fledging and non-breeding stages (Supplementary Fig. 3). In males, the home range did not change significantly during the annual cycle (*χ*^*2*^ = 4.55, *P* = 0.208; Fig. 3). A similar and stable size of home range was characteristic of both successful and unsuccessful males (Supplementary Fig. 3).

**Fig. 4:**
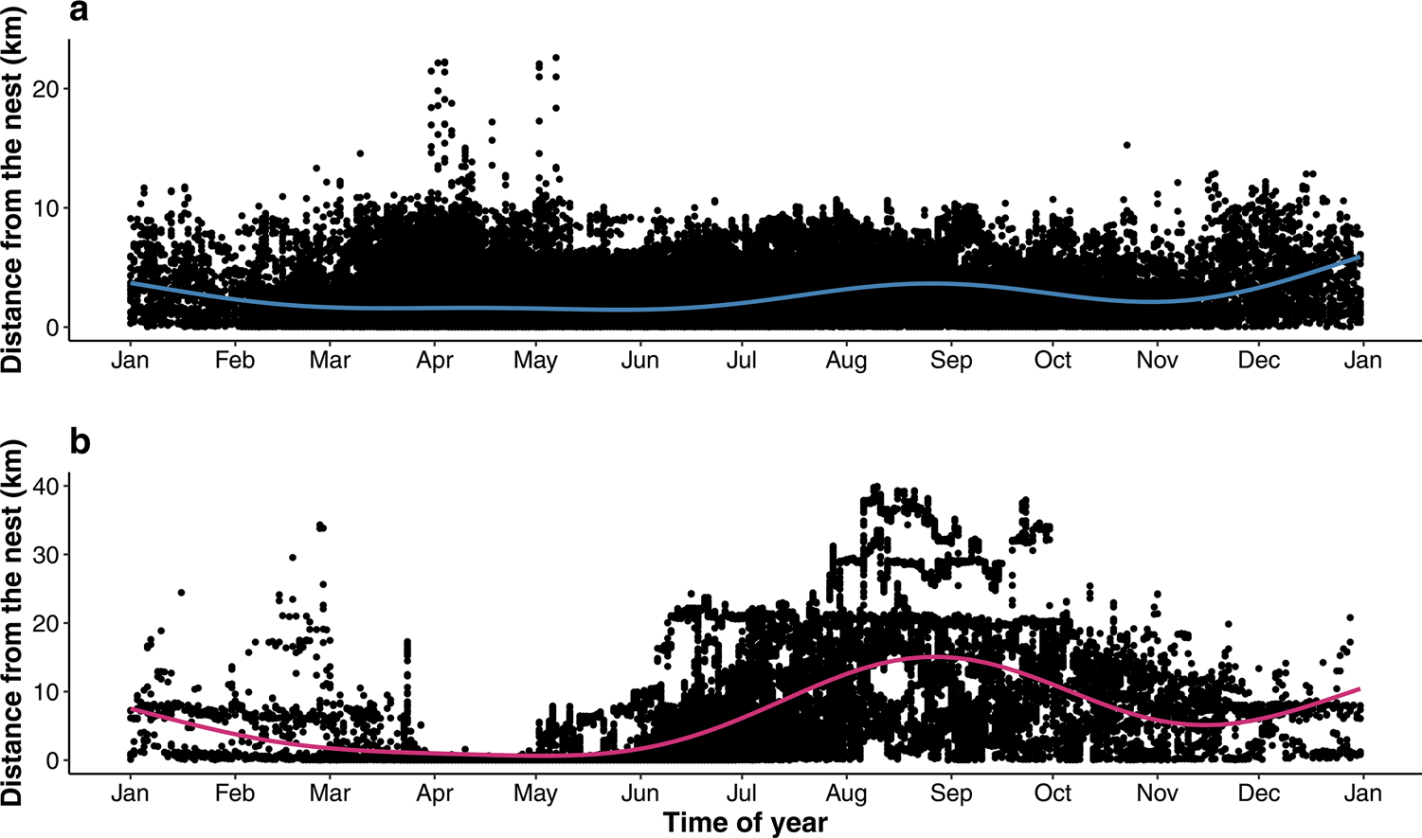
Distance of territorial male (**a**) and female (**b**) goshawks from the nest throughout the year. The line represents a GAMM-trend, surrounded by 95% confidence limits (shaded). Note the different scales on the Y-axes

Finally, distances of birds from their nests (in a given year) were measured (H4). The mean distance between GPS fixes of males from their nests was 2067 m (median 1,482); the respective distance was 7604 m (median 5,112) for females. The difference between sexes was highly significant (GAMM: edf = 8.99, *R*^*2*^_*adj*_ = 0.51, estimate_male_ = 0.247 ± 0.012 (SE), *P* = 0.001). Furthermore, males were associated with the nest throughout the year, and the distance from the nest changed only slightly after the end of the breeding stage (Fig. 4A). However, females left the breeding territory in late fledgling’s stage (August); although some females returned to the territory in September, their fidelity to the nest remained weak during the non-breeding stage (Fig. 4B).

In the two pairs tracked simultaneously for 2 years (two other pairs were tracked for a shorter time), home ranges of females overlapped with those of males completely in the incubation stage, decreased remarkably in the nestling stage (19.1 ± 21.3%), and even less in the post-fledging stage (1.7 ± 2.3%); in the non-breeding stage, the overlap was (21.2 ± 26.3%; Supplementary Table 3). For males, the overlap was negligible in the incubation stage (0.3 ± 0.2%) and lacking in the post-fledging stage; however, it was remarkable in the nestling (68.9 ± 31.4%) and non-breeding stages (73.0 ± 34.8%).

## Discussion

The home ranges of territorial goshawks tended to be smaller than those of non-territorial ones, and the ranges of successful goshawks tended to be smaller than those of unsuccessful birds. Although there was extensive variation between individuals, the individual home ranges were persistent over the years. The yearly mean home range of territorial females was remarkably larger than that of males. However, the ranges of females were significantly smaller during the incubation stage than those during the nestling, post-fledging, and non-breeding stages. Among males, the home ranges did not change significantly during the annual cycle. Finally, the males were connected to their nests throughout the year, whereas females temporarily moved away from nests. As a result, the overlap of breeding pairs decreased in the post-fledging stage (yet it increased again in the non-breeding stage). This suggested that the partners diminished competition along with the decreasing reproductive pressure.

### Factors determining home range size in male and female goshawks

The most important factor determining home range size was the sex of an individual. The mean yearly home range of male goshawks (54.4 km^2^) was almost an order of magnitude smaller than that of females (499.1 km^2^); the size difference between median home ranges was fivefold (58.3 km^2^ in males and 294.9 km^2^ in females). The difference itself is not surprising as sex is affecting home range in many species (Rolando 2002), but, interestingly, the differences between sexes have been less extensive or even contrary in previous studies on goshawks. For instance, the home range of males (51.0 km^2^) was slightly smaller than that of females (61.8 km^2^) in boreal Sweden (Widén 1989). In contrast, in northern Finland, the average home range was larger for males (70.9 km^2^) than for females (57.1 km^2^; Tornberg et al. 2006). It is worth noting that in these two Fennoscandian studies, only the winter home range was estimated, but according to our data, this is nearly equal to the annual home range. In American goshawks, home ranges of females were also smaller than those of males, for example, 38.6 km^2^ for females and 51.5 km^2^ for males during breeding time in Idaho (Moser and Garton 2019) and 12.0 km^2^ for females and 33.4 km^2^ for males in California (Blakey et al. 2020).

Notably, home ranges of both males and females were significantly larger than those detected in most earlier studies in Eurasian (Rutz 2006; Tornberg et al. 2006) or American goshawks (Boal et al. 2003, Moser and Garton 2019, Blakey et al. 2020; but see Iverson et al. 1996). Partly, the differences in home range sizes could be explained by different methodologies. First, earlier studies used radio tracking, which tends to underestimate home ranges; movements over longer distances often remain undetected (Reynolds et al. 2006), with the exception of aircraft-based radio-telemetry (Iverson et al. 1996). Second, home range size depends on the method used for estimating it. For instance, in the current study, the mean home range of females would be two times smaller if estimated using kernel density (KDE) instead of the AKDE method (unpublished data of authors). However, median values would be similar using both methods and, surprisingly, both mean and median home range of males would also be similar (unpublished data of authors). These variations, however, would not change the conclusions of the current study. Third, studies not covering the full year may underestimate the home range size as the home range size changes over seasons. However, our study shows that larger winter home ranges adequately reflect the total home range size, supporting the view that home range size depends on prey availability in the poorest season of the year (Kenward 2006).

However, the differences between the results of various studies may also be explained by ecological reasons, such as prey abundance, which depends on habitats and geographical location (Kenward 1982; Rutz et al. 2006; Tornberg et al. 2006). The goshawk is a flexible species occupying a wide array of habitats from wilderness to anthropogenic landscapes, influencing various ecological traits, including home range size. For example, the smallest home ranges have been detected in a prey-rich urban environment (Rutz 2006). Relationships between the size and habitat composition of home ranges remain beyond the scope of the current study; however, our sample of tracked birds (nest sites) covered various habitats, including forests, agricultural landscapes, and urban areas, and the distribution of tracked birds reflected proportions of the entire Estonian population. Thus, relatively large home ranges reflect generally low breeding density and probably indicate a shortage of prey in this hemiboreal region (Väli et al. 2024b).

The greater size of female home ranges was also evident in the comparison of overlapping parts of both sexes’ home ranges. Males used exclusively only around one fifth of their small home range, while most of it (78–89%) was included in their partner’s larger home range. This contrasts with patterns recorded for Montagu’s Harriers *Circus pygargus*, where males had much greater home ranges and included more than 90% of female home ranges (Krupiński et al. 2021). In that migrant species, however, home ranges were only estimated during the breeding season when females were incubating and rearing offspring, fed by the males, and thus were very limited in their space requirements. Remarkable intrapair overlap in space use (98.5%) was noted for Bonelli’s Eagles *Aquila fasciata* in Spain (Bosch et al. 2010), indicating very similar foraging patterns year-round in this sedentary, generalist species. It seems therefore that prey availability, influenced by dietary specialisation and climate severity, is causing goshawks to mitigate intrapair competition, not only through different prey selection but also by low-overlapping foraging areas.

### The effects of territoriality and reproductive performance on home range size

The largest home ranges were established by the studied non-territorial goshawks. The same pattern has been observed earlier in Sweden (Kenward et al. 1981; Widén 1985). In our study, the sample of non-territorial birds included mostly young birds. Hence, their larger home range could be attributed to being less experienced and less familiar with the local habitats and prey distribution (Widén 1985). However, food is not the only driver of space use; relatively distant movements are made in search of a mate (in females) or suitable and free territory (in males). This is a common scenario in many raptor species, where breeding adults utilise much smaller home ranges than non-breeders (Tanferna et al. 2013; Eeden et al. 2017; Mirski and Anderwald 2023), although an opposite trend was noted in Eagle Owls (*Bubo bubo*; Penteriani et al. 2015).

The lack of significant home range size difference between successful and unsuccessful birds may indicate the low reproductive cost of home range parameters in goshawks. The association between reproductive performance and home range size has been repeatedly evaluated in earlier studies, often in the context of habitat quality (i.e. prey availability), about which we unfortunately had no information. Typically, lower food resource availability in the habitat leads to larger home ranges and poorer breeding performance (e.g. Pfeiffer and Meyburg 2015, Séchaud et al. 2022). However, similarly to our study, home ranges of successful and failed Lesser Spotted Eagles did not differ from each other, although, also similar to our study, home ranges of females were significantly larger and more variable in size than those of males (Mirski et al. 2021). Although food availability is an important parameter influencing reproductive performance, it is not necessarily always associated with home range size. For example, in the Tengmalm’s owl *Aegolius funereus*, food availability influenced food provisioning rate, not home range size (Santangeli et al. 2012).

### Seasonal dynamics of home range sizes

Home ranges of Estonian females were larger than those of males throughout the year, except during incubation when females stayed at the nest. This association decreased significantly at the end of the breeding season and further after the breeding season. Our finding is similar to earlier results from North America, where home ranges of females increased two- or threefold during the non-breeding season (Moser and Garton 2019; Blakey et al. 2020). Similarly, females are less sedentary after the breeding season than males in Sweden (Widén 1985). Interestingly, several females in our study area not only enlarged their home ranges but also shifted their activity centres during the post-fledging stage, resulting in a bimodal distribution of home range (see Moser and Garton 2019 for similar results in American goshawks).

Females may require larger home ranges due to larger food demands and hunting larger prey with lower density compared to smaller prey items of males (Widén 1985; Tornberg et al. 2006). This may also force females to move away from the nest after breeding to places with a more plentiful food supply; as a result, they reach better condition by the onset of the next breeding (Widén 1985). Males, on the other hand, may stay close to the nest to compete for breeding territories, as staying nearby might be advantageous for territory holding (Widén 1985). This is supported by the continuous territorial behaviour during the non-breeding stage (Cerasoli and Penteriani 1992, unpublished data of authors). Therefore, it is advantageous for goshawk females to move across larger areas, be aware of the locations of neighbouring nests, and explore potential partners. Indeed, birds often gather in advance information about possible breeding sites before settling to breed within that area (Reed et al. 1999). This behaviour has been observed earlier in other raptors such as the Golden Eagle (*Aquila chrysaetos*; Marzluff et al. 1997) and the Lesser Spotted Eagle (*Clanga pomarina*; Meyburg et al. 2007) and may be typical also to goshawk females, whose divorce ratio, leading to breeding dispersal, is as high as 28–29% (Dietrich and Woodbridge 1994; Otterbeck et al. 2022), and must be preceded by exploratory movements.

Interestingly, several females that were not laying eggs and were not incubating (according to accelerometer data) were tightly connected with their nests during the incubation stage. The only remarkable increase of the home range during the incubation stage was noticed in a female who deserted her clutch after the death of the male. Loss of fidelity to the nest site and increase of a home range in unsuccessful females might not be an uncommon scenario in birds as it has been observed earlier in Rollers *Coracias garrulus* (Monti et al. 2024) and Mallards *Anas platyrhynchos* (Mack and Clark 2006). However, during the nestling stage, home ranges of unsuccessful female goshawks remained relatively small, whereas those of successful females increased. Supposedly, females with offspring start hunting at the end of this stage, and this is reflected in the increase of the home range. However, unsuccessful females may stay close to the nest to maintain a tight pair-bond with males; some females may even continue incubating failed broods and obtain food from males, which reduces their need to hunt and move around. In the post-fledgling stage, the average home range of unsuccessful females was larger than that of successful ones. Probably the pressure to find a partner of higher quality, or with a territory of higher quality, is stronger for unsuccessful birds. Similar results have been obtained earlier in American goshawks (Moser and Garton 2019).

We suggest that the movement of females to a different part of the home range may also be associated with the partitioning of food resources, thereby reducing competition between partners. The competition for food in hemiboreal Estonia is stronger during the non-breeding (winter) period when avian abundance is low because many potential avian prey species have migrated south, but resident goshawks stay in their breeding grounds. Similar results have been obtained in Sweden, where climatic and ecological conditions are alike; male goshawks tend to remain in the breeding area through the winter, while female goshawks tend to move away (Kenward et al. 1981; Widén 1985). In this respect, there seem to be differences between Eurasian and American goshawks. For example, no difference between male and female goshawks in (maximum) distance from nesting areas in winter was detected in Minnesota, which is rather similar to the study area in respect of climatic conditions (Boal et al. 2003).

Estonian males had similar-sized home ranges during most of the year, and they stayed close to their nests year-round. This is consistent with the earlier findings from the American goshawk (Blakey et al. 2020). Yet, the home ranges of three urban Eurasian goshawk males in Germany, which also changed in the course of the breeding season, were largest in the post-fledging stage (Rutz 2006). The tight association between males and their nest sites during the breeding season was not influenced by reproductive performance. This contrasts with several other studies in raptors demonstrating that birds that fail or skip breeding are less attached to the nest (Newton 1979). Unsuccessful Estonian males had larger home ranges only during the non-breeding stage. In Idaho, a similar pattern was observed only in females (Moser and Garton 2019). This increase may be explained by the low quality of the territory (low prey density), leading to both unsuccessful breeding and larger space requirements to fulfil energetic needs for survival during winter. In females, it would also be associated with searching for a new territory of higher quality.

## Conclusions

The Estonian goshawks in this study exhibited relatively large home ranges, potentially indicating relatively poor foraging conditions in the hemiboreal region. Females had larger home ranges than males, especially during non-breeding stages when they were not associated with nest sites. Temporal changes in home range size underscore the importance of long-term tracking for determining annual home ranges. The home ranges of males remained similar in size and were centred around their nest sites year-round, indicating strong site fidelity and the need for year-round territorial defence. Overall, we showed that space use patterns in sedentary, dietary specialist species under seasonally varying food resources are complex and may lead to different space use strategies in males and females.

## Supplementary Information

Below is the link to the electronic supplementary material.

Supplementary file1 (PDF 2163 KB)

Supplementary file2 (MP4 43107 KB)

## Data Availability

The dataset generated during the current study is publicly available in the Mendeley repository, https://data.mendeley.com/datasets/sfmsjhp4gy/2. The GPS fixes are deposited in the MoveBank repository, https://www.movebank.org/movebank/#page%3Dstudies%2Cpath%3Dstudy3274292786, and are available on request, because the raw spatial data cover the breeding sites of a species of conservation concern, protected by the Estonian Nature Conservation Act.
