# Supplementary material for: Sex-specific seasonal variation in home range size in a sedentary avian predator
Source: Oecologia. 2025 Oct 14;207(11):175. doi: 10.1007/s00442-025-05812-2 (PMC12521322; doi:10.1007/s00442-025-05812-2)
Supplement: Supplementary file 1 — Supplementary file1 (PDF 2163 KB) [file 442_2025_5812_MOESM1_ESM.pdf]

Supplementary Material

Sex-specific seasonal variation in home range size in a sedentary avian predator

Ülo Väli[1,2]*, Jaan Grosberg[1], Paweł Mirski[1,2]

Supplementary Table 1. The background information of the tracked Goshawks.

Bird id.	Sex	Age	Territoriality	Reproductive performance	Tracking period*	No. of days tracked	No. of fixes	Fate* and the pairbond of the bird.
17319	Female	2cy	Territorial	Unsuccessful	18.01.22-27.06.22	161	524	Perished
171095	Male	Ad	Territorial	7× successful	22.03.19-31.08.25	2355	30972	Alive
190703	Male	Ad	Territorial	3× successful	25.03.22-27.06.24	826	26594	Perished, partner of 190730
190706	Male	Ad	Territorial	Unsuccessful	03.08.22-24.10.22	83	516	Device stopped data transmission
190710	Male	Ad	Territorial	4× unsuccessful	04.06.19-21.03.23	1387	6378	Perished
190710_2	Male	2cy	Nonterritorial		15.10.23-31.08.2025	687	13767	Alive
190723	Male	2cy	Nonterritorial		14.01.22-10.04.22	87	894	Perished
190723_2	Male	Ad	Territorial	2× successful, 1×unsuccessful	14.04.23-26.08.25	865	9395	Alive
190725	Male	2cy	Nonterritorial		19.01.22-03.07.22	166	7950	Perished
190725_2	Male	Ad	Territorial	Successful	05.04.23-20.10.23	199	2253	Device stopped data transmission
190728	Male	Ad	Territorial	3× unsuccessful, 1× successful	18.03.22-31.08.25	1263	50336	Alive; partner of 212347
190730	Female	2cy	Territorial	Successful	23.03.22-30.06.22	100	2297	Perished; partner of 190703
190730_2	Female	Ad	Territorial	Successful	05.04.23-23.09.23	172	4319	Device stopped data transmission
191323	Female	Ad	Territorial	Successful	19.06.19-25.11.19	160	6322	Device stopped data transmission
191324_3	Female	2cy	Territorial	Successful	18.06.20-16.09.20	91	3325	Perished
191324_5	Female	Ad	Territorial	2× successful	18.03.22-24.11.23	617	11212	Perished
201716	Female	2cy	Nonterritorial		29.03.24-05.01.25	282	13760	Perished
201717	Female	Ad	Territorial	2× successful	24.03.22-30.07.23	494	6552	Device stopped data transmission; partner of 212340

212340	Male	Ad	Territorial	4× successful	25.03.22-31.08.25	1256	66982	Alive, partner of 201717
212341	Male	Ad	Territorial	Unsuccessful	23.03.22-22.04.22	31	1247	Perished, partner of 212346
212342	Male	Ad	Territorial	3× unsuccessful	18.03.22-20.07.24	863	53251	Perished
212346	Female	Ad	Territorial	Unsuccessful	23.03.22-19.11.22	242	7925	Perished, partner of 212341
212347	Female	Ad	Territorial	2× unsuccessful	18.03.22-05.10.23	567	11960	Perished; partner of 190728
212347_2	Female	Ad	Territorial	2× successful	28.02.24-31.08.25	550	22016	Alive
223914	Male	Ad	Territorial	Successful	26.08.23-17.12.23	66	264	Device stopped data transmission

* As of 15.09.2025

Supplementary Table 2. Home range sizes and overlapping parts of the ranges in pairs where both partners were tracked. Note that in the last two pairs, one partner was tracked only for a short period.

IDs of the pair	Year	Female 90% AKDE (sq km)	Male 90% AKDE (sq km)	Overlapping area (sq km)	Proportion of overlap in males (%)	Proportion of overlap in females (%)
201717 / 212340	2022	385.00	48.31	48.31	100	12.55
201717 / 212340	2023	334.90	43.50	43.50	100	13.68
212347 / 190728	2022	637.38	49.69	44.46	89.47	6.97
212347 / 190728	2023	494.43	46.12	43.04	93.32	8.76
190730 * / 190703	2022	1.48	15.04	1.38	9.16	93.24
212346 / 212341 **	2022	1725.70	63.47	63.47	100	3.68

* - female tracked only between 23 March and 30 June 2022

** - Male tracked only between 23 March and 22 April 2022

Supplementary Table 3. Home range sizes and overlapping parts of the ranges during four stages of the annual cycle in pairs where both partners were tracked throughout the year. N – number of studied 'goshawk-years'

Stage	N	Female 90% AKDE (sq km)	Male 90% AKDE (sq km)	Overlapping area (sq km)	Proportion of overlap in males (%)	Proportion of overlap in females (%)
Incubation	5	8.0 ± 17.6	31.4 ± 19.1	0.1 ± 0.1	0.3 ± 0.2	100 ± 0
Nestling	5	257.0 ± 160.0	31.9 ± 10.1	23.6 ± 14.0	68.9 ± 31.4	19.1 ± 21.3
Post-fledging	4	124.4 ± 76.4	78.1 ± 54.4	2.9 ± 4.4	6.7 ± 9.2	1.7 ± 2.3
Nonbreeding	6	615.2 ± 576.8	45.2 ± 15.4	35.5 ± 19.9	73.0 ± 34.8	21.2 ± 26.3

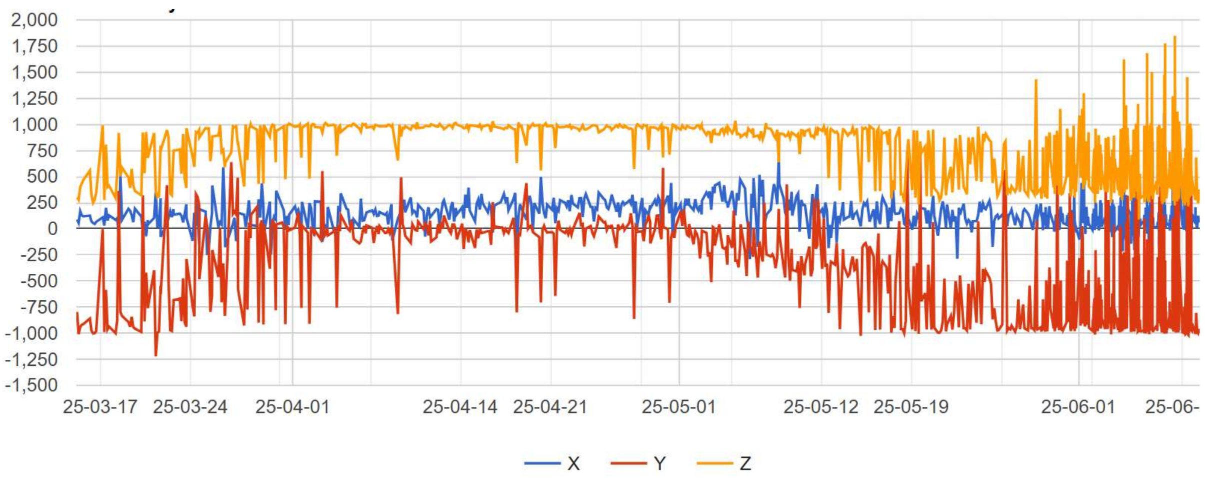

Supplementary Figure 1. An example accelerometer graph indicating the behavioural changes of a female goshawk during breeding period according to its posture.

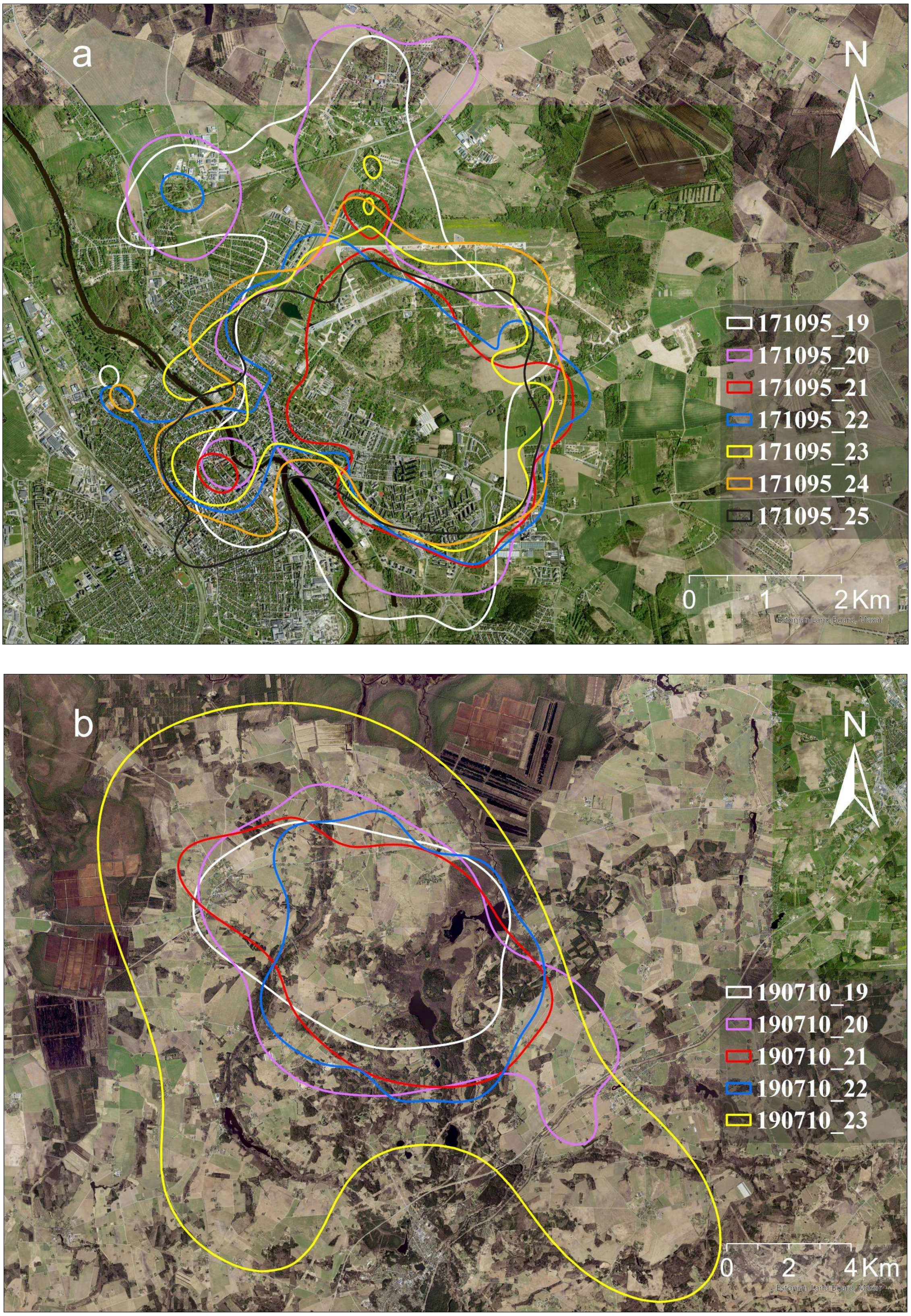

Supplementary Figure 2. Annual home ranges (90% AKDE) of the two males during seven (171095 in 2019–2025, **a**) and five years (190710 in 2019–2023, **b**) respectively.

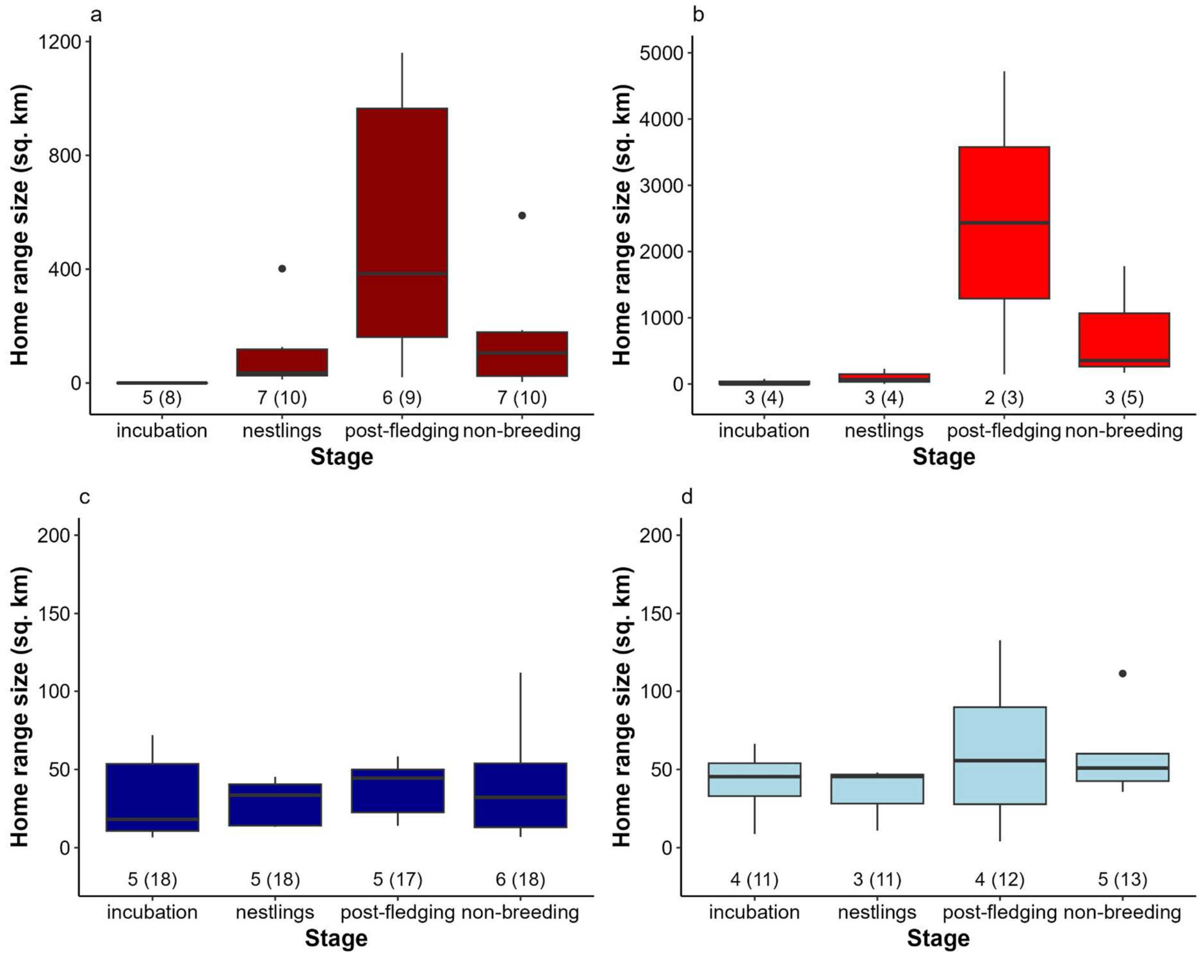

Supplementary Figure 3. Home range sizes (90% AKDE) of successful (A) and unsuccessful female (B) and successful (C) and unsuccessful male (D) Goshawks during the annual cycle stages. The bold line indicates the median, the box shows quartiles, the whiskers indicate the extreme data points within 1.5× the interquartile range from the quartile boundaries and dots are data points beyond that range. Numbers of studied individuals (and goshawk-years in brackets) are presented on the x-axis. Note the different scales of Y-axes.

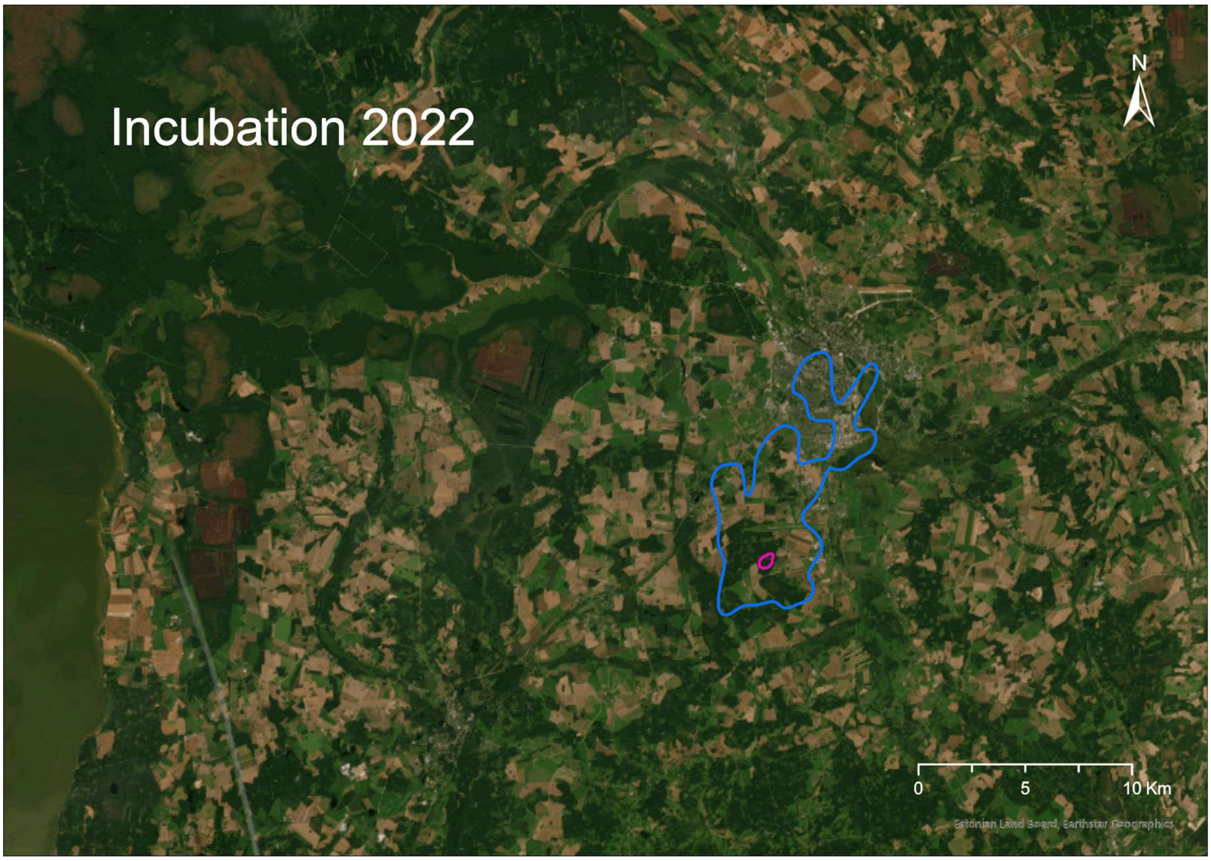

Supplementary Video 1. 90% AKDE Home ranges of the pair 212347 / 190728 (range of the female as pink and that of male as blue line) during four annual cycle phases in 2022 and 2023. In both years, breeding was not initiated (eggs were not laid).
